# Identifying relevant asymmetry features of EEG for emotion processing

**DOI:** 10.3389/fpsyg.2023.1217178

**Published:** 2023-08-17

**Authors:** Fatima Islam Mouri, Camilo E. Valderrama, Sergio G. Camorlinga

**Affiliations:** Department of Applied Computer Science, University of Winnipeg, Winnipeg, MB, Canada

**Keywords:** emotion recognition, electroencephalogram, affective computing, brain hemisphere asymmetry, logistic regression, interpretable predictive models

## Abstract

The left and right hemispheres of the brain process emotion differently. Neuroscientists have proposed two models to explain this difference. The first model states that the right hemisphere is dominant over the left to process all emotions. In contrast, the second model states that the left hemisphere processes positive emotions, whereas the right hemisphere processes negative emotions. Previous studies have used these asymmetry models to enhance the classification of emotions in machine learning models. However, little research has been conducted to explore how machine learning models can help identify associations between hemisphere asymmetries and emotion processing. To address this gap, we conducted two experiments using a subject-independent approach to explore how the asymmetry of the brain hemispheres is involved in processing happiness, sadness, fear, and neutral emotions. We analyzed electroencephalogram (EEG) signals from 15 subjects collected while they watched video clips evoking these four emotions. We derived asymmetry features from the recorded EEG signals by calculating the log ratio between the relative energy of symmetrical left and right nodes. Using the asymmetry features, we trained four binary logistic regressions, one for each emotion, to identify which features were more relevant to the predictions. The average AUC-ROC across the 15 subjects was 56.2, 54.6, 51.6, and 58.4% for neutral, sad, fear, and happy, respectively. We validated these results with an independent dataset, achieving comparable AUC-ROC values. Our results showed that brain lateralization was observed primarily in the alpha frequency bands, whereas for the other frequency bands, both hemispheres were involved in emotion processing. Furthermore, the logistic regression analysis indicated that the gamma and alpha bands were the most relevant for predicting emotional states, particularly for the lateral frontal, parietal, and temporal EEG pairs, such as FT7-FT8, T7-T8, and TP7-TP8. These findings provide valuable insights into which brain areas and frequency bands need to be considered when developing predictive models for emotion recognition.

## 1. Introduction

Emotion recognition is an active research area in affective computing, neuroscience, and psychology. As emotions elicit specific behavioral responses, researchers often employ techniques to monitor bodily reactions and expressions to recognize different emotional states (Kop et al., 2011). One reliable method for identifying these reactions is through the use of neuroimaging techniques, such as Electroencephalography (EEG) and functional Magnetic Resonance Imaging (fMRI), which can help monitor brain activity (Rangayyan, 2002).

Brain activity reflects the magnetic and electrical activity experienced by neurons during emotional perception and regulation (Gunes and Pantic, 2010; Valderrama and Ulloa, 2012). However, research has demonstrated that this electrical activation is not uniform throughout the brain, and each hemisphere is associated with different behavior. Specifically, neuroscientists have proposed two models to explain this brain asymmetry (Alves et al., 2008). The first model, known as ‘the right-hemisphere dominance of emotions', suggests that the right hemisphere is more involved in processing emotions than the left hemisphere (Borod et al., 1998; Demaree et al., 2005). In contrast, the second model, known as ‘the valence lateralization', assumes lateralization based on the type of emotions, where the right hemisphere processes negative emotions, and the left hemisphere processes positive emotions (Ahern and Schwartz, 1985; Davidson, 1995). Nonetheless, recent neuroscience studies have challenged these asymmetrical models by reporting that the brain exhibits dynamic behavior and a bilateral activation (Morawetz et al., 2020; Stanković and Nešić, 2020; Palomero-Gallagher and Amunts, 2022). For instance, Stanković and Nešić (2020) reported that, initially, the brain displays a right-biased pattern for emotional perception, but after experiencing psychological conditions, such as stress or demanding emotional tasks, the distribution of brain activity is altered and redistributed across both hemispheres.

In addition to the distinction between hemispheres in emotion processing, neural frequency bands (delta, theta, alpha, beta, and gamma) also play distinct roles (Park et al., 2011). Indeed, researchers have focused on the alpha band (8–12 Hz) to analyze hemispheric asymmetries for different emotions using a methodology known as frontal alpha asymmetry (FAA) (Briesemeister et al., 2013). Specifically, FAA compares the electrical activity in the alpha band of the right and left hemispheres in the frontal and prefrontal areas, detecting more cortical activity in one hemisphere when its electrical activity is lower than in the other hemisphere (Gevins et al., 1997; Allen et al., 2004). The foundation of FAA relies on the fact that EEG power is inversely related to activity, indicating that lower power means more cortical activity (Lindsley and Wicke, 1974). For example, Zhao et al. (2018) used the FAA methodology to analyze EEG signals collected from subjects watching videos evoking tenderness and anger, reporting that the left hemisphere had more cortical activity when the subjects perceived tenderness, whereas the right hemisphere had more cortical activity when watching angry videos. Unlike the alpha band, beta and gamma frequency bands do not show asymmetrical behavior during emotion processing (Davidson et al., 1990). These bands exhibit similar patterns when processing emotions, such as reduced power in beta and gamma bands across the cortex during happiness and increased power in frontal regions of both hemispheres during anger Aftanas et al. (2006).

Inspired by the brain asymmetry for processing emotion, previous researchers have attempted to improve the accuracy of machine learning models to predict emotional states (Huang et al., 2012; Ahmed and Loo, 2014; Li et al., 2022; Sajno et al., 2022). To that aim, researchers have proposed features that capture the brain's asymmetrical behavior in emotion processing. These features aim to measure differences in EEG signals extracted from symmetrical positions in both hemispheres. The most commonly employed asymmetry feature type is based on the power spectrum, where the difference or ratio of electrical activity between two EEG nodes is computed (Huang et al., 2012; Duan et al., 2013; Aris et al., 2018). Although nonlinear features, such as fractal dimension, correlation dimension, or RQA analysis, have been extensively used for emotion recognition (Liu and Sourina, 2014; Yu et al., 2016; Li et al., 2018; Soroush et al., 2018, 2019, 2020; Yang et al., 2018), only one previous study has used fractal dimension to derive asymmetry indexes to classify emotions (Liu et al., 2010).

Among the studies using spectral features to reflect brain asymmetry, Pane et al. (2019) used a random forest to classify four emotions (happy, sad, relaxed, and angry) from five EEG pairs (*F*7-*F*8, *T*7-*T*8, *C*3-*C*4, *O*1-*O*2, and *CP*5-*CP*6) under four different scenarios. The first scenario used the brain activity of both hemispheres to train the random forest. In contrast, the second scenario used only the information from the right hemisphere to train the random forest, whereas the third scenario used only the information from the left hemisphere. The fourth scenario used information from the right hemisphere to predict negative emotions (sad and angry) and information from the left hemisphere to predict positive emotions (happy and relaxed). The authors reported that the fourth scenario using the *T*7-*T*8, *C*3-*C*4, and *O*1-*O*2 electrode pairs obtained the best performance for predicting emotions, thus suggesting that the inclusion of emotional lateralization is beneficial for emotion recognition approaches. Similarly, Zheng and Lu (2015) used asymmetry features calculated by taking the difference and ratio of EEG electrodes from the left and right hemispheres to train a support vector machine and a deep belief networks (DBNs) model to recognize positive, neutral, and negative emotions. The authors found that the performance of asymmetry features varied across the frequency bands, achieving the best performance for the beta and gamma bands. Li et al. (2021) further explored the discrepancy between the brain hemispheres for processing emotions by developing a deep learning model that used the horizontal and vertical streams from the left and right EEG electrodes as input. Their model yielded an accuracy between 58.13 and 74.43% for detecting four emotional states across 15 subjects. Moreover, the authors reported that the EEG nodes located in the frontal region (*FP*1-*FP*2, *AF*3-*AF*4, *F*7-*F*8, *F*5-*F*6, *F*3-*F*4, *F*1-*F*2, *FT*7-*FT*8, *FC*5-*FC*6, *FC*3-*FC*4, and *FC*1-*FC*2) were the most relevant for the classification of emotions.

Recent studies using deep learning models have also leveraged brain asymmetry to extract features reflecting asymmetric hemisphere patterns to classify emotional states. Yan et al. (2022) proposed a hemispheric asymmetry measurement network (HAMNet), which is an end-to-end network capable of automatically learning discriminant features for emotion classification tasks. Ding et al. (2022) employed a multi-scale convolutional neural network to extract both temporal and spatial features for emotion recognition. Their approach involved applying multi-scale one-dimensional convolutional kernels to the EEG channels of each hemisphere, thereby extracting comprehensive global spatial representations. Luan et al. (2022) introduced a long short-term memory (LSTM) layer to extract asymmetrical patterns from both hemispheres across different frequency bands, improving the performance of cross-subject emotion classification. Ahmed et al. (2022) expanded the exploration of asymmetry between the left and right hemispheres to encompass the entire cortex. In detail, the authors generated a 62-square matrix by taking the difference between the differential entropy of each pair of EEG nodes. This matrix was fed into a CNN model to automatically extract features. The results showed that the automated features obtained from the CNN model outperformed manually extracted features, such as the difference and ratio of differential entropy between left and right EEG nodes, in accurately classifying positive, negative, and neutral emotions.

Until now, previous research studies have mostly focused on using the asymmetry of emotion processing to build more accurate predictive models. However, little research has been conducted to explore how machine learning models can help to identify associations between brain asymmetry, frequency bands, and emotional states. The complex models used for training the predictive models, such as deep neural networks or support vector machines, are challenged by limited interpretability because they are unable to explain their predictions (Arrieta et al., 2020). Moreover, most of these studies have used a subject-dependent approach (Li et al., 2022), in which the predictive models are trained and tested using EEG signals of the same subject, thus limiting the capacity for generalizing neuronal patterns among different subjects. Although we note that brain activity varies among human beings due to factors such as age, sex, and health status (Kudielka et al., 2009; Heimann et al., 2016), using a subject-independent approach may provide insights on which EEG channels pair are more relevant for processing positive or negative emotions.

In this study, we explore how brain asymmetry is involved in processing happiness, sadness, fear, and neutrality using a subject-independent approach. We conducted two experiments on a dataset containing electroencephalogram (EEG) signals from 15 subjects collected while watching video clips evoking the four emotions. We used statistical hypothesis tests and logistic regression, an interpretable predictive model, to identify which ratios between the left and right EEG nodes and which frequency bands are more relevant to predict each type of emotion. Our approach was able to identify which EEG channels and frequency bands are more relevant to predict happiness, sadness, fear, and neutrality across subjects. Our results reveal that the pairs *T*7-*T*8, *FT*7-*FT*8, and *TP*7−*TP*8 in the gamma band and the pair *FT*7-*FT*8 in the alpha band were relevant to discriminate between happiness, sadness, and fear. The findings provide valuable insights into the specific areas of the brain that contain essential information for recognizing different emotional states, thus helping to design emotion recognition approaches.

## 2. Materials and methods

**Figure 1:**
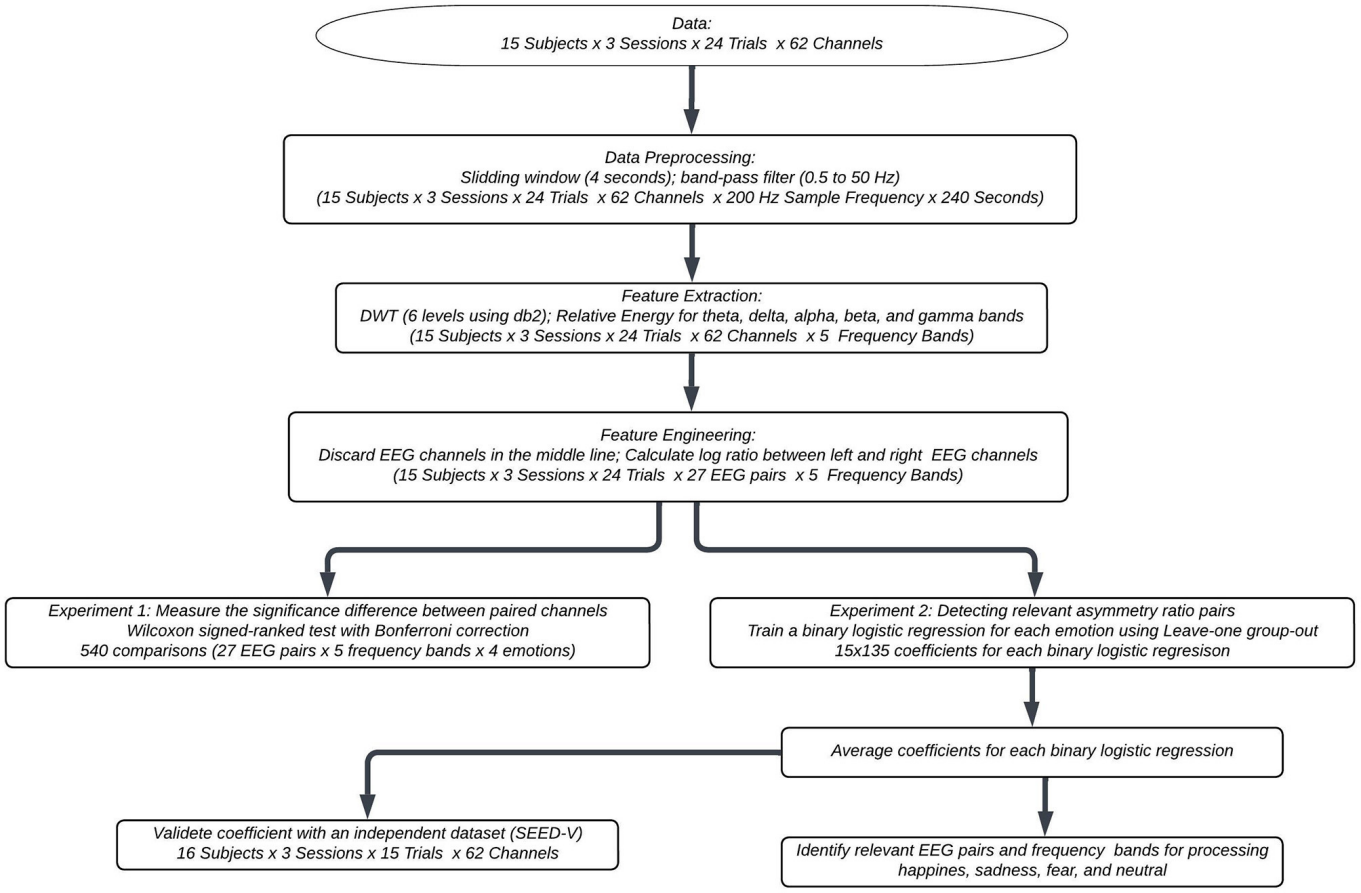
Flowchart of the methodology of our study.

Figure 1 shows the flowchart of the proposed methodology. The subsequent subsections elaborate on the specific details of each component.

### 2.1. Data set

This work used the SEED-IV database (Zheng et al., 2018). The dataset consists of EEG signals recorded from 15 healthy, right-handed subjects aged between 20 and 24, with eight female participants. Each subject participated in three distinct sessions during which they watched a total of 72 videos. These videos were carefully selected based on a consensus among 44 raters, ensuring that they were capable of eliciting emotions such as happiness, fear, sadness, and neutrality. The subjects viewed the videos across three separate sessions, with each session consisting of 24 video clips. In every session, the number of video clips for each emotion was six. While watching the video clips, the subject's EEG signals were recorded using 62 EEG channels at a sampling rate of 1,000 Hz. After recording, the EEG signals were downsampled to 200 Hz. In total, 72 EEG signals (one per video clip) were recorded for each subject.

### 2.2. Preprocessing

The collected EEG signals were initially resampled at 200 Hz to ensure a Nyquist frequency of 100 Hz. As EEG signals are susceptible to artifacts and noise stemming from eye blinking and various physiological and non-physiological processes, it is advisable to employ signal filtering techniques to mitigate their impact on classification. Several methods have been proposed for filtering EEG signals. Advanced methods include the use of supervised machine learning to classify noise segments and applying recurrent neural networks to remove such noises (Ghosh et al., 2023). However, although this method has shown to be promising, it necessitates the availability of class labels (noisy vs. non-noisy) for each EEG segment. Alternative methods based on the spectrum domain involve using EEG signals collected in a relaxed state to adjust the spectrum of EEG responses evoked by emotional stimuli, thereby removing inter-subject differences in EEG patterns (Ahmed et al., 2023). Simpler methods for removing artifacts and noises include the use of band-pass filters (Li et al., 2022). However, it is crucial to note that band-pass filtering may not eliminate all artifacts, as eyeblink frequencies fall below 4 Hz, while muscular artifacts exhibit frequencies higher than 13 Hz (Boudet et al., 2008).

Since noise labels or EEG data collected in a relaxed state were unavailable in this study, we employed a Butterworth bandpass filter with a frequency range of 0.5–50 Hz to process the EEG signals. This filter ensured to keep the frequency range associated with delta, theta, alpha, beta, and gamma brainwaves. The filtered EEG signals were divided into segments using a non-overlapping window of 4 seconds. The window duration of 4 seconds was chosen to achieve a frequency resolution of 0.5 Hz ($2\times \frac{1}{4s}$), allowing the detection of the delta frequency band (0.5–4 Hz).

### 2.3. Feature extraction

We used the discrete wavelet transform (DWT) to extract spectral features from the filtered 4-second window. Because previous studies have indicated that the Daubechies 2 (db2) wavelet function is suitable for processing EEG signals for classification purposes due to its scaling properties and resemblance to EEG's spike-wave patterns (Güler and Übeyli, 2005; Subasi, 2007; Gandhi et al., 2011; Tong et al., 2018), we used the db2 function to decompose EEG signals into six levels. The decomposition process using db2 as the wavelet mother is presented in Table 1. The detail coefficients from the second to the sixth level corresponded to the gamma, beta, alpha, theta, and delta bands.

**Table 1:** Frequency decomposition level for the Discrete Wavelet Transform (DWT).

**Level**	**Frequency range (Hz)**	**Coefficients**	**Sub-band**
1	100–50	d1	
2	50–25	d2	Gamma
3	25–12.5	d3	Beta
4	12.5–6.25	d4	Alpha
5	6.25–3.125	d5	Theta
6	3.125–1.5625	d6	Delta
6	1.5625–0	a6	

We used these detail coefficients of the gamma, beta, alpha, theta, and delta band to calculate the percentage of energy content at each frequency band, a feature that has shown potential for emotion recognition approaches (Valderrama and Ulloa, 2014; Krisnandhika et al., 2017; Valderrama, 2021). In detail, the the percentage of energy for each band was calculated as:


(1) $$\begin{array}{l} \begin{array}{l} E_{ch,b}=\frac{\sum_{i=1}^{N_{c}}d_{ch,b}{\left(i\right)}^{2}}{E_{Total}} \end{array} \end{array}$$

where *d* is the *i*^*th*^ detail coefficient of the *ch*_*th*_ channel (e.g., *Fp*_1_, *O*_2_, *T*_1_) and the *b*_*th*_ band (delta, theta, alpha, beta, or gamma), *N*_*c*_ denotes the number coefficient at the band *b*, and *E*_*Total*_ is the sum of the squared coefficients over all levels.

### 2.4. Feature engineering for the asymmetry analysis

Since our focus in this study was to explore the involvement of the hemisphere asymmetry in processing emotions, we only considered the 54 EEG channels located on the right and left hemispheres, discarding the 8 EEG channels on the center line. We then paired the 54 EEG channels by mapping those channels positioned equidistantly from the center line. Table 2 shows the 27 mapped EEG pairs.

**Table 2:** Paired 54 EEG channels from the left and right hemispheres.

**Pair**	**1**	**2**	**3**	**4**	**5**	**6**	**7**	**8**	**9**
Left	FP1	AF3	F1	F3	F5	F7	FT7	T7	TP7
Right	FP2	AF4	F2	F4	F6	F8	FT8	T8	TP8
**Pair**	**10**	**11**	**12**	**13**	**14**	**15**	**16**	**17**	**18**
Left	FC1	FC3	FC5	C1	C3	C5	CP1	CP3	CP5
Right	FC2	FC4	FC6	C2	C4	C6	CP2	CP4	CP6
**Pair**	**19**	**20**	**21**	**22**	**23**	**24**	**25**	**26**	**27**
Left	P1	P3	P5	P7	PO3	PO5	PO7	O1	CB1
Right	P2	P4	P6	P8	PO4	PO6	PO8	O2	CB2

A total of 27 channels from the left hemisphere were paired with the 27 channels from the right hemisphere. EEG channels were paired so that the distance to the midline was equal.

To compute an asymmetry feature, we adapted the rational asymmetry index based on power spectrum density (Huang et al., 2012; Duan et al., 2013; Aris et al., 2018) for the wavelet energy percentage. Specifically, we calculated the asymmetry feature by taking the natural logarithm of the ratio between the energy in the left and right hemispheres, as follows:


(2) $$\begin{array}{l} ratio_{ch_{x},ch_{y},b}=\log\frac{E_{ch_{x},b}}{E_{ch_{y},b}} \end{array}$$

where *E*_*c*_*h*__*x*_, *b*_ and *E*_*c*_*h*__*y*_, *b*_ were the energy of the left EEG channel *x* and the right EEG channel *y* for the frequency band *b*.

### 2.5. Asymmetry analysis

We conducted two experiments to explore which bands and channel pairs were more involved in emotion processing. In the first experiment, we performed statistical hypothesis tests to determine whether there were significant differences between the energy of the 27 EEG pairs. In the second experiment, we trained a logistic regression model for each emotion (neutral, sadness, happiness, and fear) using the asymmetry-ratio features (Equation 2) to identify which bands and channel pairs were more important for predicting each type of emotion.

#### 2.5.1. Experiment 1: comparing electrical activity in the right and left hemispheres

In order to compare differences between the left and right hemispheres when processing emotions, we applied the logarithmic properties to Equation 2 to express the ratio as a difference as:


(3) $$\begin{array}{l} {\text{Δ}}_{ch_{x},ch_{y},b}=\log E_{ch_{x},b}-\log E_{ch_{y},b} \end{array}$$

where *E*_*c*_*h*__*x*_, *b*_ and *E*_*c*_*h*__*y*_, *b*_ were the energy of the left EEG channel *x* and the right EEG channel *y* for the frequency band *b*.

We then used the Wilcoxon signed-rank test to compare whether the distribution of the logarithmic difference between the EEG channel pairs (see Table 2) was symmetric about zero. The null and alternative hypotheses were defined as follows:


(4) $$\begin{array}{l} wilcoxon({\text{Δ}}_{ch_{x},ch_{y},b})=\left\{ \begin{array}{l} \eta_{{\text{Δ}}_{ch_{x},ch_{y},b}}=0\text{ null hypothesis}\\ \eta_{{\text{Δ}}_{ch_{x},ch_{y},b}}\neq 0\text{ alternative hypothesis} \end{array}\right. \end{array}$$

where η_Δ_*c*_*h*__*x*_, *ch*_*y*_, *b*__ was the median of the logarithmic energy difference in the frequency band *b* between the left EEG electrode *x* and the right EEG electrode *y*.

Considering that we performed 540 hypothesis tests for the 27 pairs, four emotions, and five frequency bands, we corrected the significance value for the hypothesis tests using Bonferroni correction (α = 0.05/540 = 0.00009) to reduce the probability of type I error (false positives).

#### 2.5.2. Experiment 2: detecting relevant asymmetry ratio pairs

To further investigate which asymmetry ratios between the EEG channel pairs were most relevant for predicting each type of emotion, we used a one-vs.-all approach to train a logistic regression model for each emotion. This approach involved training a separate logistic regression model for each emotion, with the corresponding emotion class labeled as “1” and the other emotions labeled as “0”. The reason for using a one-vs.-all approach instead of a multinomial logistic regression was that the individual binary logistic regressions allowed us to obtain a vector of coefficients that associated which ratios between the left and right EEG channels and which frequency bands were most relevant for predicting each specific emotion of interest. In contrast, a multinomial logistic regression would select an emotion as a reference and provide three coefficient vectors comparing the odds of a sample being of each emotion relative to the reference emotion.

We used the “leave-one group-out” (LOGO) cross-validation technique to train each binary logistic regression, ensuring that our models were subject-independent. This technique guaranteed that no EEG signals from a single subject were present in both the training and test sets during each iteration. As we had data from 15 subjects, we performed 15 iterations, with each iteration using the EEG samples from one subject as the test set, and the samples from the remaining 14 subjects forming the training set.

At each iteration of the LOGO cross-validation, we trained the logistic regression models using the training set and evaluated the performance of our models using eight different metrics on the test set. These metrics included sensitivity, specificity, positive predictive value (PPV), negative predictive value (NPV), F1-score, geometric mean (G-mean), the area under the Receiver Operating Characteristics (AUC-ROC) curve, and the area under the precision-recall curve (AUC-PR). Sensitivity measures the proportion of true positives (samples correctly classified as belonging to the target emotion) out of all the actual target emotion samples. Specificity measures the proportion of true negatives (samples correctly classified as not belonging to the target emotion) out of all the actual non-target emotion samples. PPV measures the proportion of true positives out of all the samples classified as belonging to the target emotion, while NPV measures the proportion of true negatives out of all the samples classified as not belonging to the target emotion. The F1-score is the harmonic mean of precision and recall, while the G-mean is the square root of the product of sensitivity and specificity. The AUC-ROC measures the overall ability of the model to distinguish between target and non-target emotion samples, while the AUC-PR measures the trade-off between precision and recall for different classification thresholds. Table 3 shows a mathematical representation of the metrics used to assess the performance of binary logistic regression models.

**Table 3:** Metrics used to measure the performance of the binary logistic regression models.

**Metric**	**Equation**
Sensitivity (or recall)	*TP*/(*FN*+*TP*)
Specificity	*TN*/(*FP*+*TN*)
Positive predictive value (or precision) (PPV)	*TP*/(*FP*+*TP*)
Negative predictive value (NPV)	*TN*/(*FN*+*TN*)
F1-score	2 × (*precision*×*recall*)/(*precision*+*recall*)
Geometric mean (G-mean)	$\sqrt{sensitivity\times specificity}$
Area under the receiver operating characteristic curve (AUC-ROC)	$\overset{1}{\underset{0}{\int }}TPR\left(t\right)d\left(FPR\left(t\right)\right)$
Area under the precision-recall curve (AUC-PR)	$\overset{1}{\underset{0}{\int }}precision\left(t\right)d\left(recall\left(t\right)\right)$

The following acronyms are used: TP, true positive; TN, true negative; FP, false positive; FN, false negative; TPR, true positive rate; FPR, false positive rate. For the AUC-ROC, *d*(*FPR*(*t*)) denotes the differential change in the false positive rate at the threshold *t*. Similarly, for AUC-PR, *d*(*recall*(*t*)) represents the differential change in the recall at the threshold *t*.

In total, we had 135 asymmetry features (Equation 2), which were derived from the 27 EEG pairs multiplied by five frequency bands. Since developing subject-independent emotion recognition approaches is challenging due to the poor generalizability of features across subjects, we implemented two pre-processing feature steps to train the logistic regression model.

First, in order to determine which features were more relevant for the emotion of interest, we conducted an ANOVA F-value analysis. This analysis allowed us to assess whether the means of each feature were significantly different across the two classes. A higher F-value indicated a larger difference in means between classes compared to the variation within each class. Using the p-values associated with the F-value of each feature, we selected those features that were statistically significant (*p-*value < 0.05).

Second, to further remove irrelevant asymmetry features and to avoid overfitting, we used regularization, a technique that has shown to be effective for subject-independent emotion recognition approaches (Li et al., 2018). Thus, we applied elastic net regularization (Zou and Hastie, 2005) to train each logistic regression at each iteration of the LOGO cross-validation. The best hyperparameters for the l1-ratio (ϕ) and the regularization strength (*C*) were optimized using nested-cross validation on the training set. The grid search used nested-cross validation was defined by ϕ ∈ {0, 0.1, 0.2, , ..., 0.8, 0.9, 1} and *C* ∈ {10^−3^, 10^−2^, ..., 10^2^}. All logistic regression models were fitted without an intercept (i.e., setting *w*_0_ = 0) to ensure that the predictions depended only on the features.

At each of the 15 iterations of the LOGO cross-validation, the logistic regression generated a coefficient for each of the 135 asymmetry features. We stored these 135 logistic regression coefficients at each iteration. Once the LOGO cross-validation was complete, we averaged the logistic regression coefficients across the iterations, thus obtaining a set of coefficients for each type of emotion. These sets of coefficients indicated which features (i.e., asymmetry log-ratios between EEG pairs and frequency bands) were most relevant for predicting each type of emotion.

#### 2.5.3. Finding relevant EEG channels for predicting emotions

To identify relevant EEG channels in processing emotions, we conducted two analyses with the logistic regression coefficients. First, we identified the most relevant features for the binary prediction of the four logistic regressions by identifying the five highest and the five lowest average coefficients.

For our second analysis, we used the logistic regression coefficients to generate brain topography maps. To that aim, we leveraged the fact that the trained logistic regression models predict the class of a sample using the following equation:


(5) $$\begin{array}{l} \hat{y}=\frac{1}{1+e^{-\left(\sum_{i=1}^{135}\omega_{i}f_{i}\right)}}, \end{array}$$

where ŷ is the probability that the sample belongs to the emotion of interest, ω_*i*_ is the logistic regression coefficient associated with the *i*_*th*_ feature (*f*_*i*_), which corresponds to the log-ratio of the relative energy between the left and right EEG channels (Equation 2).

The 135 coefficients correspond to the combination of the 27 EEG channel pairs (Table 2) and the five frequency bands (delta, theta, alpha, beta, and gamma). By expanding the log-ratio of the features, the summation in the exponent of Equation 5 can be rewritten as:


(6) $$\begin{array}{l} \sum_{i=1}^{135}\omega_{i}f_{i}=\sum_{j=1}^{27}\sum_{k=1}^{5}\omega_{j\cdot k}\log\frac{E_{ch_{x_{j}},b_{k}}}{E_{ch_{y_{j}},b_{k}}}\\ \;=\omega_{1}\log\frac{E_{ch_{x_{1}},b_{1}}}{E_{ch_{y_{1}},b_{1}}}+...+\omega_{135}\log\frac{E_{ch_{x_{27}},b_{5}}}{E_{ch_{y_{27}},b_{5}}}\\ \;=\omega_{1}\log E_{ch_{x_{1}},b_{1}}-\omega_{1}\log E_{ch_{y_{1}},b_{1}}+...\;\\ \;+\omega_{135}\log E_{ch_{x_{27}},b_{5}}-\omega_{135}\log E_{ch_{y_{27}},b_{5}}, \end{array}$$

where *E*_*c*_*h*__*x*_*j*__, *b*_*k*__ and *E*_*c*_*h*__*y*_*j*__, *b*_*k*__ were the energy of the left EEG channel *x* and the right EEG channel *y* for *j*_*th*_ log-ratio at the frequency band *b*_*k*_.

The prediction of the emotion of interest can be determined based on the sign of the summation in Equation 6. If the summation is positive, the sample is predicted to belong to the emotion of interest. Conversely, if the summation is negative, the sample is predicted not to belong to the emotion of interest. Therefore, when the logistic coefficient ω_*i*_ is positive, the energy of the left EEG channel *x* at the frequency band *b*_*k*_ (*E*_*c*_*h*__*x*_*j*__, *b*_*k*__) is directly associated with the emotion of interest. In contrast, in such cases, the energy of the right EEG channel *y* at the frequency band *b*_*k*_ (*E*_*c*_*h*__*y*_*j*__, *b*_*k*__) is inversely related to the emotion prediction. On the other hand, when the logistic coefficient ω_*i*_ is negative, the opposite relationship holds: the energy of the left EEG channel is inversely related to the emotion, whereas the energy of the right EEG channel is directly related to the emotion. Table 4 summarizes the four cases we used to establish the relationship between the emotions, the features *f*, and the logistic regression coefficients, ω.

**Table 4:** Relationships between logistic regression coefficients, EEG channel energy, and emotions.

**ω_*i*_**	**Feature**	**Interpretation**
+	*E*_*c*_*h*__*left*_, *b*_	Relative energy of the left channel at frequency band *b* was directly related to the emotion
		*E*_*c*_*h*__*right*_, *b*_	Relative energy of the right channel at frequency band *b* was inversely related to the emotion
−	*E*_*c*_*h*__*left*_, *b*_	Relative energy of the left channel at frequency band *b* was inversely related to the emotion
		*E*_*c*_*h*__*right*_, *b*_	Relative energy of the right channel at frequency band *b* was directly related to the emotion

The first column displays the sign of the logistic regression coefficient (+ or –). The second column represents the features used for fitting the logistic regression: the energy of the EEG channel at frequency band *b*. The last column interprets the association between the coefficient, feature, and the emotion.

### 2.6. Validation with external dataset

To evaluate the generalization capacity of the average coefficients in predicting emotions, we conducted a performance test using an independent dataset, specifically the SEED-V dataset (Liu et al., 2021). The SEED-V dataset comprises EEG data collected from subjects while they watched video clips representing five different emotions: happy, sad, fear, disgust, and neutral. The study involved 16 participants (6 male and 10 female) who each watched 15 movie clips (3 clips per emotion) across 3 sessions. EEG signals were recorded from 62 EEG channels at a sampling rate of 1,000 Hz.

To maintain consistency with the dataset used to train the logistic regression models, we excluded the EEG samples corresponding to disgust and focused solely on samples associated with happiness, sadness, neutral, and fear. We processed the EEG signals following the same methodology used for SEED-IV signals (refer to subsection 2.2), and computed the asymmetry features by calculating the logarithmic difference between the left and right EEG nodes, as described in subsection 2.3.

For each of the four emotions, we fitted a logistic regression with the coefficients as the average coefficients obtained for the 15 subjects of the SEED-IV. We then calculated the AUC-ROC value for each of the 16 subjects of the SEED-V dataset.

## 3. Result

### 3.1. Comparing electrical activity in the right and left hemispheres

**Figure 2:**
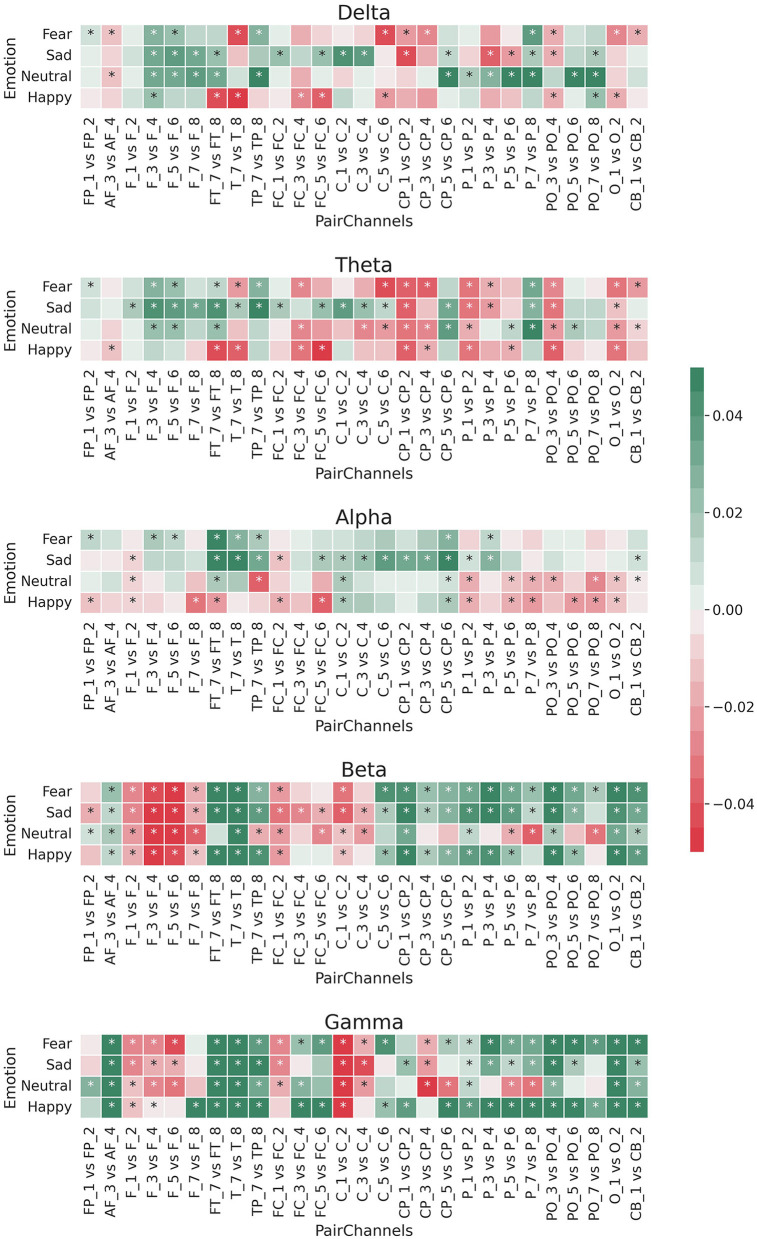
Median of logarithmic wavelet energy difference (Equation 3) between the paired EEG electrodes for the five band frequencies: delta, theta, alpha, beta, and gamma. *means that the difference was statistically significant [Bonferroni corrected *p*-value 0.00009 (0.05/540); Wilcoxon rank sum test].

Figure 2 shows the logarithmic difference (Equation 3) between the medians of the energy of the 27 paired EEG channels for each of the five frequency bands. Most of these differences were significant [Wilcoxon rank sum test with Bonferroni correction; *p-*value 0.00009 (0.05/540)], most occurring at the beta and gamma frequency bands.

For the beta and gamma bands, the left channels had more relative energy than the right channels for each brain region except the frontal area (*F*_1_/*F*_2_, *F*_3_/*F*_4_, *F*_5_/*F*_6_, and *F*_7_/*F*_8_). This pattern was the opposite for the theta and delta bands, in which the right channels resulted in more relative energy for EEG channels outside the frontal area. In the case of the alpha frequency band, there was a difference among the emotions. Specifically, for sad and fear, the log differences of the relative energy between the left and right channels resulted more in positive values (green colors), while for happy and neutral emotions, those log differences were mostly negative (red colors).

### 3.2. Predicting emotions using relevant asymmetry ratio pairs

Appendix Tables 5–8 present the performance metrics for the binary logistic regression models across the 15 subjects. The sensitivity (the accuracy for correctly detecting samples from the emotion of interest) and the specificity (the accuracy for correctly detecting samples not from the emotion of interest) exhibited variability across the subjects, with a mean sensitivity between 51.0 and 52.4%, and a mean specificity between 51.0 and 59.2%. The G-mean, a metric that combines these two metrics while considering data imbalance, obtained a mean value of 52.7, 51.7, 50.1, and 52.6% for the neutral, sad, fear, and happy models, respectively. The ROC-AUC score showed a similar classification capacity for the models, with a mean value of 56.2% for neutral, 54.6% for sad, 51.6% for fear, and 58.4% for happy. The NPV, which indicates the precision in detecting samples that were not from the emotion of interest, exhibited the best performance across the four models, with an average value between 75.1 and 82.1%. This result suggests that the models were accurate in detecting samples that were not from the emotion of interest. The mean performance of the eight metrics for the training and testing sets were similar, indicating low overfitting during the training process.

**Figure 3:**
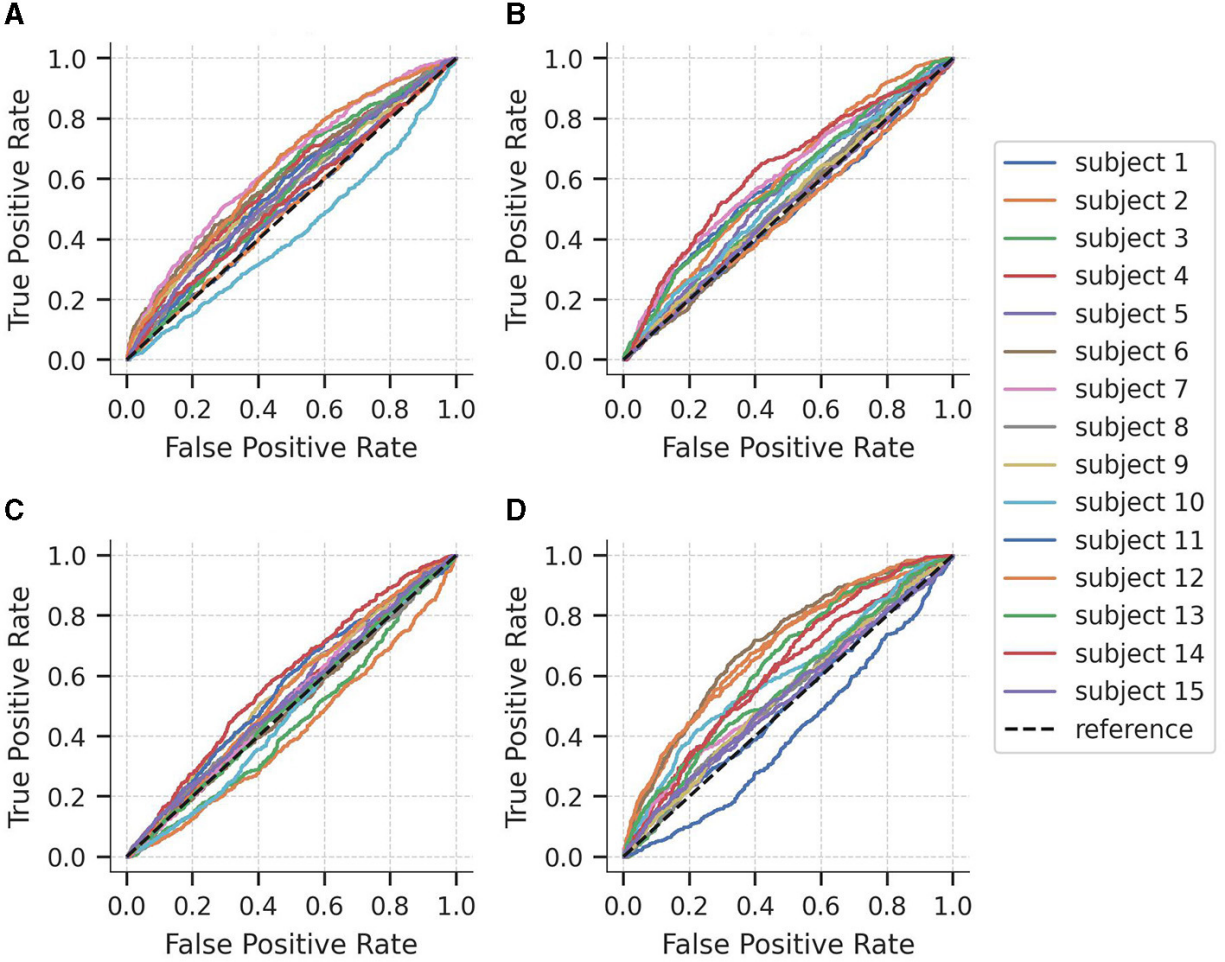
Area under the curve of the Receiver Operating Characteristics (AUC-ROC) curves for each subject of the SEED-IV dataset for predicting **(A)** neutral vs. non-neutral, **(B)** sad vs. non-sad, **(C)** fear vs. non-fear, and **(D)** happy vs. non-happy.

To further explore the performance of the models at different classification thresholds, Figure 3 displays the AUC-ROC curves for each subject of the SEED-IV dataset across the four binary logistic regression models. The results exhibit considerable variability in performance across subjects, with AUC-ROC values ranging from approximately 50 to 60%. Among the models, the classification of happy vs. non-happy demonstrates the highest discriminative capacity, whereas the fear vs. non-fear model exhibits the lowest ability to differentiate between samples belonging to the class and non-class categories.

### 3.3. Relevant EEG channels for predicting emotions

**Figure 4:**
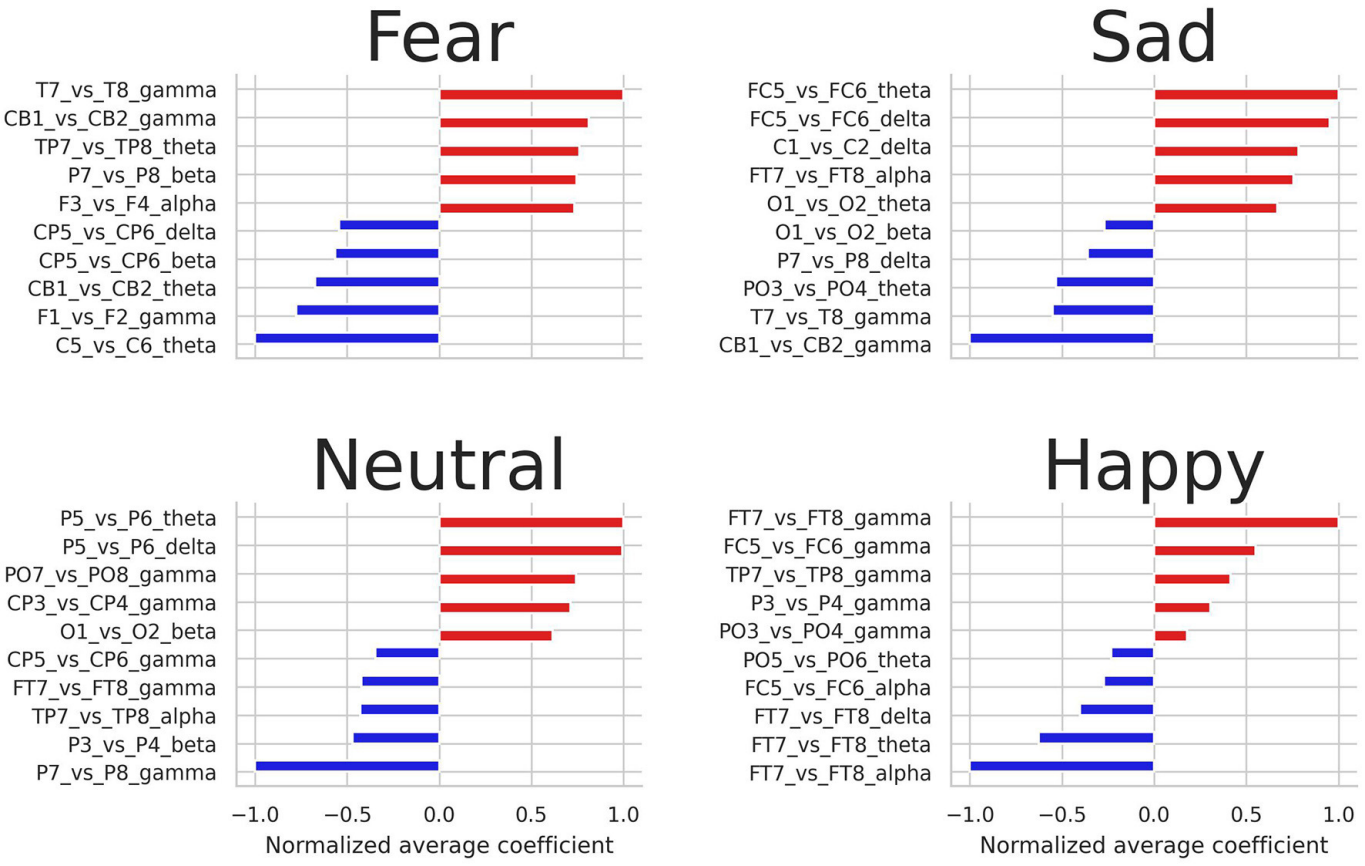
Highest and lowest five average coefficients for predicting fear, sadness, neutral, and happiness.

Figure 4 shows the highest and lowest normalized average coefficients for predicting fear, sadness, neutral, and happiness. Notably, the gamma frequency band emerges as the most influential in distinguishing between the four emotional states, with energy log-ratios from this band serving as crucial features for predicting the four emotional states. Additionally, asymmetry features extracted from the theta frequency band were also relevant. Regarding EEG pairs, the pairs *FT*7-*FT*8, *TP*7-*TP*8, and *FC*5-*FC*6 were among the most crucial pairs to predict the four emotions.

**Figure 5:**
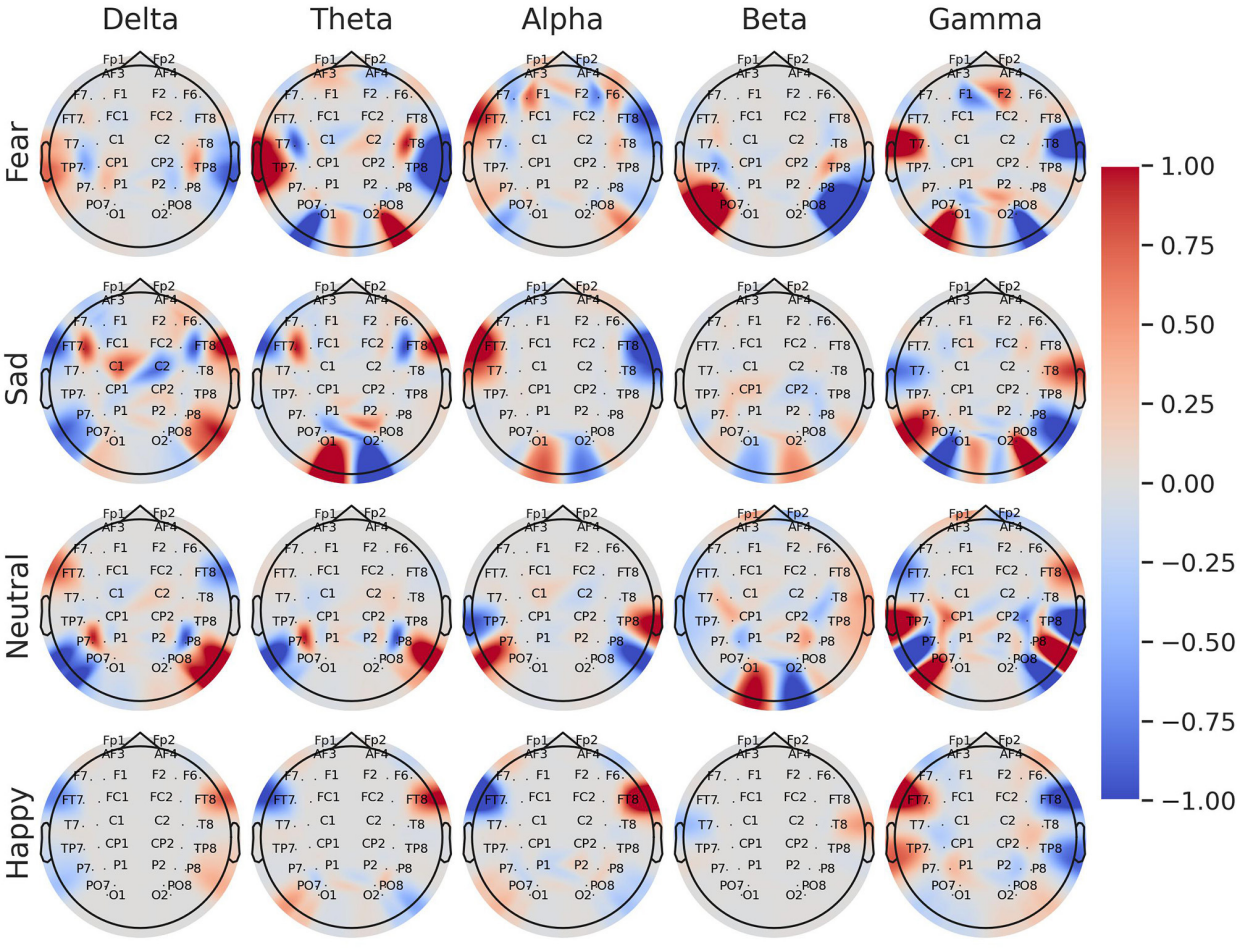
Brain topography of the normalized average coefficients for logistic regression models trained with asymmetry log-ratio features for each individual class: fear, sadness, neutrality, and happiness. The color tones represent the associations between the energy of the EEG channel and the emotion. Red tones indicate a direct association between the energy of the EEG channel and the emotion, whereas blue tones indicate an inverse association.

Figure 5 shows a brain topography with the average coefficients of the logistic regression models for each emotion and frequency band. Most of the associations between the emotions and the energy of EEG channels were located in the lateral frontal, temporal, and parietal areas. Specifically, in the gamma band, greater relative energy values of *T*7 were directly related to fear and inversely related to happiness and sadness. In contrast, greater relative energy values of the left EEG channel *FT*7, *TP*7, and *PO*3 were directly related to happiness and inversely related to fear. Notably, in the alpha band, higher values of energy in the channel *FT*7 were directly related to sadness and fear, while inversely related to happiness. The lower frequency bands, theta, and delta, mostly indicated direct and inverse relationships for the negative emotions (sad and fear).

### 3.4. Validation with external dataset

**Figure 6:**
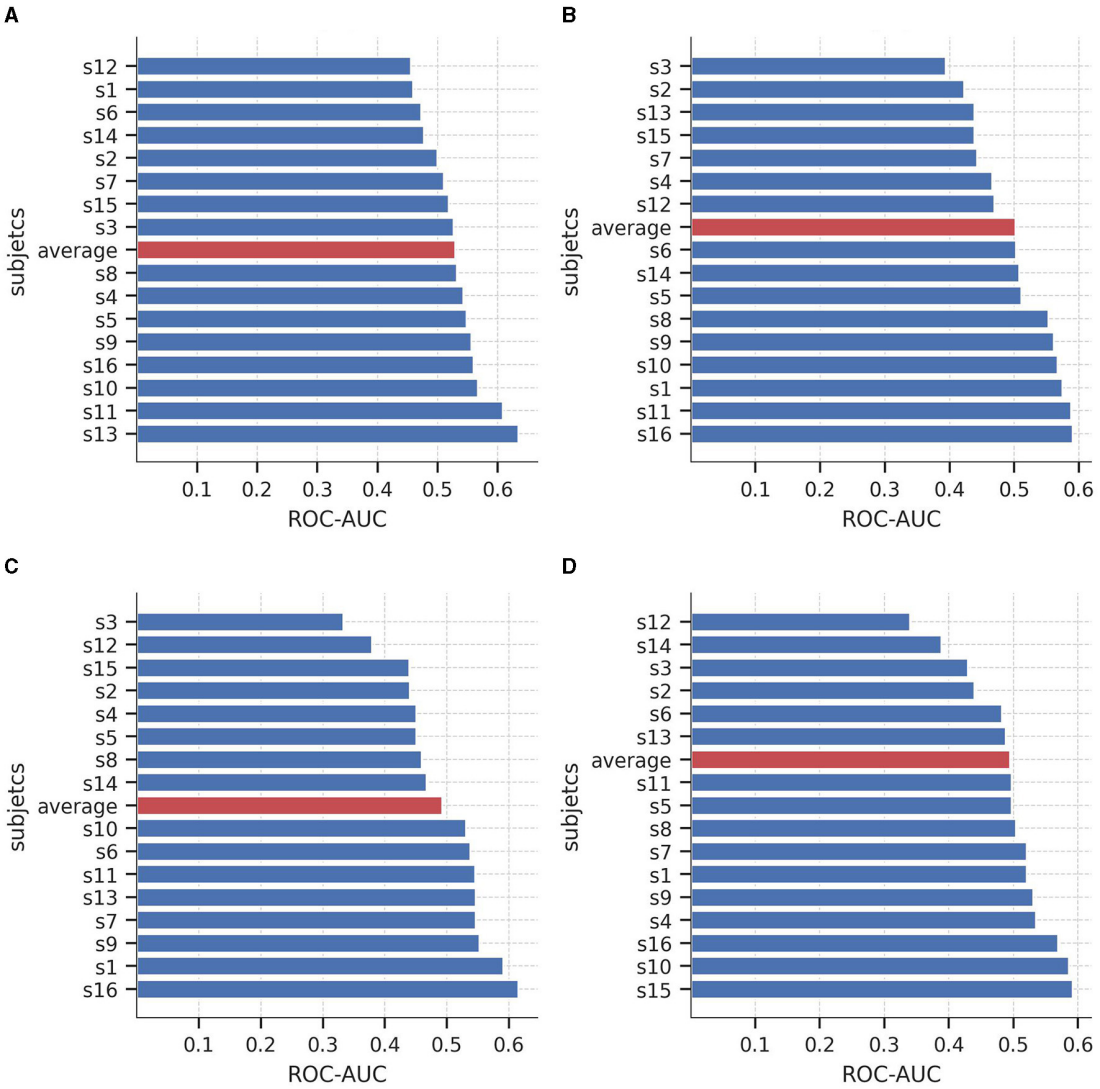
Area under the curve of the Receiver Operating Characteristics (AUC-ROC) value for the 16 subjects of the SEED-V dataset for predicting **(A)** neutral vs. non-neutral, **(B)** sad vs. non-sad, **(C)** fear vs. non-fear, and **(D)** happy vs. non-happy. Red bars shows the average AUC-ROC across the subjects.

Figure 6 illustrates the AUC-ROC of the average coefficients when evaluated on subjects from an independent unseen dataset, SEED-V. Consistent with the findings from SEED-IV, the AUC-ROC yielded in an average range of around 0.5, with a variable performance across the subjects. Specifically, in the sad and happy classes, nine out of the 16 subjects achieved an AUC-ROC of 0.5 or higher. In the neutral and fear classes, eight subjects attained an AUC-ROC of 0.5 or higher. These results emphasize the individual differences in classification performance among subjects when detecting emotions from EEG signals.

## 4. Discussion

Brain complexity and high variance across subjects challenge the mapping between EEG electrical activity and emotions (Li et al., 2022). These mappings could benefit from examining the asymmetry in emotional processing between the left and right hemispheres of the brain (Alves et al., 2008). In this study, we explored the utilization of asymmetry features to identify the EEG channels and frequency bands that are more relevant for predicting happiness, sadness, fear, and neutrality across subjects. To that aim, we employed the DWT to extract the relative energy of each frequency band, and then we calculated the log-ratio of electrodes located in symmetrical positions. To our knowledge, this is the first study to utilize DWT-extracted relative energy to describe the brain's asymmetry in processing happiness, sadness, fear, and neutrality.

Our results indicate that brain hemispheres and frequency bands participated differently in processing happiness, sadness, fear, and neutrality. Unlike the “right-dominance” (Borod et al., 1998; Demaree et al., 2005) and the “valence lateralization” (Ahern and Schwartz, 1985; Davidson, 1995) models, we found that the involvement of the hemisphere is more related to the frequency bands. Only in the alpha frequency band (8–12 Hz), we found lateralization when comparing a positive emotion (happiness) with negative emotions (sadness and fear). The distinct behavior shown in the alpha band between the positive (happy) and negative (sad and fear) emotions is consistent with the FAA analysis (Briesemeister et al., 2013). Higher electrical activation on the frontal right hemisphere channels (*FP*2, *F*4, and *F*6) for processing happiness indicates a large cortical resource allocation on the left hemisphere. Therefore, the left hemisphere is more involved in processing positive emotions. Similarly, higher activation on the left frontal channels (*FP*1, *F*3, and *F*5) for sadness and fear indicates more involvement of the right hemisphere to process negative emotions. This similarity is explained by the fact that the FAA is the logarithmic difference between the power of the two hemispheres, while our asymmetry feature is the logarithmic difference between the energy of the two hemispheres. Therefore, given that power is the amount of energy divided by time, there is a natural correlation between FAA and our asymmetry feature for the alpha band.

For the other frequency bands, both hemispheres were involved in processing emotions. The left hemisphere played a more significant role in processing emotions at higher frequency bands (gamma and beta), whereas the right hemisphere was more involved at lower frequency bands (theta and delta). This bilateral activation aligns with recent findings in neuroscience, indicating dynamic behavior and balanced activation in both hemispheres (Morawetz et al., 2020; Stanković and Nešić, 2020; Palomero-Gallagher and Amunts, 2022). As highlighted by Stanković and Nešić (2020), the brain initially demonstrates a right bias, but when emotional tasks are introduced, the electrical activity becomes distributed between both hemispheres. Considering that the data used in this study involved video clips lasting approximately two minutes each, it is expected that the activation of both hemispheres would increase throughout the recording session, as emotional stress was experienced.

The binary logistic regression models yielded valuable insights into the significance of frequency bands and EEG nodes in predicting emotions. The average coefficients obtained from the four trained models indicated the involvement of all frequency bands in emotion prediction, with the gamma band being particularly relevant. In particular, asymmetry features extracted from the gamma band in the temporal, frontal, and parietal areas (*FT*7-*FT*8, *T*7-*T*8, and *TP*7-*TP*8) emerged as relevant features for predicting emotions. The significance of this frequency band and its association with cortex areas can be visually demonstrated through brain topography (refer to Figure 5), where an inverse relationship between fear and happiness is observed for the EEG pairs *FT*7-*FT*8, *T*7-*T*8, and *TP*7-*TP*8 within the gamma band. Additionally, the topography reveals that EEG nodes located at the sides of the scalp hold the greatest relevance across all frequency bands in predicting emotional states. These findings offer valuable insights into the brain regions involved in emotional processing and may have important implications for developing more accurate and efficient models for emotion recognition using EEG signals.

Regarding the comparison between negative and positive emotions, our findings suggest that happiness is more opposite to fear than sadness. In detail, our logistic regression analysis showed a contradictory behavior between the coefficients for the EEG pairs in the gamma band for predicting happiness and fear. Specifically, our results suggest that higher relative energy at the *PO*3 node in the gamma band increases the likelihood of a subject experiencing happiness and decreases the likelihood of experiencing fear. In contrast, higher values of the EEG node *T*7 in the gamma band increase the probability of a subject experiencing fear and decrease the probability of experiencing happiness. Similarly, for the alpha band, higher positive values of the EEG node *FT*7 are directly related to identifying fear and inversely related to identifying happiness. Given that happiness and fear are emotions with high arousal, whereas sadness is an emotion with low arousal, it seems that the asymmetry lateralization is more pronounced for emotions that have opposite valence but higher arousal levels.

In regard to previous work, our findings also support the importance of the gamma and beta bands in processing emotions (see Figure 4), as previously reported by Zheng and Lu (2015). Additionally, our logistic regression analysis identified the ratios *T*7/*T*8, *FT*7/*FT*8, and *FC*5/*FC*6 as relevant EEG pairs to discriminate between positive and negative emotions, which is consistent with Pane et al. (2019), who found that brain activity extracted from the pairs *T*7-*T*8, *C*3-*C*4, and *O*1-*O*2 was important for classifying different emotional states. Finally, our results are in line with Zheng et al. (2018), who also reported that neural information from the EEG nodes located in the frontal region, such as *FP*1-*FP*2, *FT*7-*FT*8, and *FC*5-*FC*6, is one of the most relevant for predicting different emotional states.

It is important to acknowledge that the performance of the logistic regression models varied across subjects, with AUC-ROC values ranging from 41 to 70% for all 15 subjects across the four emotions. This notable inter-subject variability in the metrics highlights the difficulty of subject-independent approaches, which are inherently challenging due to the substantial EEG variability observed between individuals (Arevalillo-Herráez et al., 2019; Chen et al., 2021). Nonetheless, the limited over-fitting observed between the training and test sets indicates that the logistic regression models were able to capture some extent of emotional processing patterns. Interestingly, when utilizing the average coefficients to predict emotions in an independent dataset (SEED-V), a similar performance in terms of AUC-ROC values was achieved, suggesting that the extracted patterns may generalize when applied to EEG signals obtained from new subjects.

We also note that our accuracy rates were lower than those obtained by the deep learning models (Li et al., 2021). However, as reported in Li et al. (2021), it is expected that non-deep learning models achieved a lower performance for subject-independent predictions in the SEED-IV dataset, with an accuracy between 31 and 37% for regression models and SVM. Although we cannot directly compare with previous studies using regression models and SVM because we trained an individual model rather than a multi-class model, our performance is within the expected range for non-deep learning models attempting subject-independent approaches (Takahashi et al., 2004; Chanel et al., 2011).

One notable advantage of our approach over more accurate but complex deep learning models (Zheng and Lu, 2015; Li et al., 2021) is the interpretability provided by logistic regression models. This interpretability enabled us to successfully identify significant asymmetry relationships between EEG pairs and frequency bands associated with the processing of happiness, sadness, fear, and neutrality. Furthermore, the use of a subject-independent approach, combined with the interpretability of our model, facilitated the identification of shared patterns among the 15 subjects included in our dataset.

The lower metric performances might be a consequence of using a logistic regression model, a predictive model with high interpretability but lower performance rates than deep learning models (Arrieta et al., 2020). Future studies could attempt to solve this issue by using predictive models that have a balance between accuracy and interpretability, such as fuzzy modeling. This might be useful in improving the performance of emotion recognition as well as preserving interpretability.

One limitation of our study is that although we used an independent dataset to assess our findings, both datasets are from similar populations. In particular, both datasets (SEED-IV and SEED-V) were collected from students of Shanghai Jiao Tong University. As EEG data exhibit different brain patterns among individuals due to factors such as gender, culture, and genetics (Hamann and Canli, 2004), our results may not apply to individuals of different cultures. For instance, previous studies have reported that differences between Western and Asian cultures can affect the performance of emotion recognition approaches (Bradley et al., 2001). Nevertheless, despite the fact that both SEED-IV and SEED-V were collected at the same location, individuals of both datasets are mutually exclusive, allowing a fair validation of the results presented in our study.

Another area for improvement of our study is the absence of exploration into advanced artifact removals methods, such as recurrent networks (Ghosh et al., 2023) and spectrum adjustment (Ahmed et al., 2023). EEG signals are commonly contaminated by various artifacts, including muscle, and eye-blinking artifacts, which can significantly impact the accuracy of emotion classification. To address this limitation, future improvements could involve integrating pre-processing techniques that outperform traditional frequency filters in effectively eliminating artifacts caused by eye blinking and muscle movement. By incorporating these advanced artifact removal techniques, it is possible to reduce the effect of confounding factors on emotion recognition. Further research should focus on exploring these methods to improve the robustness and reliability of our findings.

## 5. Conclusion

This study aims to explore the association between asymmetry EEG pairs and frequency bands in the processing of happiness, sadness, fear, and neutrality. The findings revealed that the gamma and alpha bands in the lateral frontal, temporal, and parietal regions (T7-T8, FT7, FT7-FT8, and TP7-TP8) played a crucial role in distinguishing and predicting positive and negative emotions. Regarding the neuroscience models, we observed valence lateralization in the alpha band, with positive emotions predominantly processed in the left hemisphere and negative emotions in the right hemisphere. However, for the other frequency bands, both hemispheres were found to be involved in the processing of emotions. These outcomes provide valuable insights into the specific brain regions and frequency bands that should be considered when developing predictive models for emotion recognition. By considering these findings, future research can focus on leveraging these relevant features to enhance the accuracy and robustness of emotion classification models.

## Data availability statement

Publicly available datasets were analyzed in this study. This data can be found here: https://bcmi.sjtu.edu.cn/~seed/seed-iv.html#.

## Ethics statement

Ethical review and approval was not required for the study on human participants in accordance with the local legislation and institutional requirements. Written informed consent from the patients/participants or patients/participants legal guardian/next of kin was not required to participate in this study in accordance with the national legislation and the institutional requirements.

## Author contributions

CV and SC are the supervisors of FM. FM and CV performed the experiments and contributed to the writing of the manuscript. SC acquired the data used for the experiments. All authors reviewed and approved the final manuscript.
