# Supplementary material for: Identifying relevant asymmetry features of EEG for emotion processing
Source: Front Psychol. 2023 Aug 17;14:1217178. doi: 10.3389/fpsyg.2023.1217178 (PMC10469865; doi:10.3389/fpsyg.2023.1217178)
Supplement: Supplementary file 1 [file Data_Sheet_1.pdf]

APPENDIX

Table 5. Sensitivity (Sen.), specificity (Spe.), Positive Predictive Value (PPV), Negative Predictive Value (NPV), F1-score, Geometric Mean (G-Mean), the area under the curve of the Receiver Operating Characteristics (AUC-ROC) and the area under the precision-recall curve (AUC-PR) for predicting *Neutral* across the 15 subjects.

Subject	Sen (%)		Spe (%)		PPV (%)		NPV (%)	
	Train	Test	Train	Test	Train	Test	Train	Test
1	53.2	52.7	58.1	50.7	32.0	28.4	77.0	74.3
2	59.1	64.6	57.5	35.2	34.0	27.0	79.1	72.8
3	53.2	52.4	57.3	62.8	31.6	34.3	76.7	78.0
4	56.9	68.4	56.1	46.4	32.5	32.2	77.8	79.8
5	52.8	30.4	58.6	74.7	32.1	30.8	77.0	74.3
6	56.5	46.2	56.5	70.5	32.5	36.7	77.8	77.9
7	56.9	71.5	55.8	46.6	32.3	33.2	77.7	81.5
8	57.0	46.3	56.6	61.5	32.8	30.8	78.0	75.5
9	56.8	56.0	56.1	54.0	32.5	31.1	77.8	76.8
10	58.0	40.3	56.9	48.8	33.3	22.6	78.5	68.8
11	53.3	72.6	57.6	35.6	31.8	29.5	76.9	77.8
12	58.3	50.4	56.6	66.2	33.3	35.7	78.5	78.3
13	57.5	48.2	56.5	56.4	32.9	29.1	78.2	74.6
14	56.4	31.1	57.1	74.0	32.8	30.8	77.9	74.3
15	53.1	55.2	58.1	53.5	32.0	30.6	76.9	76.3
Mean	55.9	52.4	57.0	55.8	32.6	30.8	77.7	76.1

Subject	F1-score (%)		G-Mean (%)		AUC-ROC (%)		AUC-PR (%)	
	Train	Test	Train	Test	Train	Test	Train	Test
1	40.0	36.9	55.6	51.7	58.0	51.5	34.2	27.9
2	43.2	38.1	58.3	47.7	62.1	50.5	37.2	27.4
3	39.6	41.4	55.2	57.3	57.6	60.9	33.6	36.5
4	41.4	43.8	56.5	56.4	59.4	59.4	35.2	36.1
5	40.0	30.6	55.6	47.6	58.1	53.8	33.9	31.8
6	41.3	40.9	56.5	57.0	59.4	60.9	35.1	40.1
7	41.2	45.3	56.3	57.7	59.0	64.7	34.8	41.1
8	41.6	37.0	56.8	53.4	59.8	55.6	35.8	31.2
9	41.3	40.0	56.5	55.0	59.5	57.3	35.3	35.9
10	42.3	29.0	57.5	44.3	60.8	42.6	36.8	23.8
11	39.9	41.9	55.4	50.8	57.9	56.4	34.1	30.6
12	42.3	41.8	57.4	57.8	60.7	63.6	35.8	39.5
13	41.9	36.3	57.0	52.2	59.9	54.6	36.0	29.6
14	41.5	30.9	56.8	48.0	59.8	53.4	35.8	30.6
15	39.9	39.3	55.5	54.3	57.9	57.1	33.9	32.9
Mean	41.2	38.2	56.5	52.7	59.3	56.2	35.2	33.0

Table 6. Sensitivity (Sen.), specificity (Spe.), Positive Predictive Value (PPV), Negative Predictive Value (NPV), F1-score, Geometric Mean (G-Mean), the area under the curve of the Receiver Operating Characteristics (AUC-ROC) and the area under the precision-recall curve (AUC-PR) for predicting *Sad* across the 15 subjects.

Subject	Sen (%)		Spe (%)		PPV (%)		NPV (%)	
	Train	Test	Train	Test	Train	Test	Train	Test
1	53.6	51.2	56.7	64.4	31.7	35.1	76.5	77.9
2	55.4	52.3	59.3	60.8	33.8	33.3	78.0	77.3
3	56.0	74.8	57.6	28.3	33.1	28.1	77.7	75.0
4	55.6	51.8	57.6	50.2	32.9	28.1	77.6	73.6
5	58.3	41.9	52.3	65.9	31.4	31.5	77.0	75.1
6	56.0	41.6	59.8	57.0	34.3	26.6	78.4	72.2
7	55.9	51.0	58.9	66.1	33.8	36.0	78.1	78.2
8	56.8	57.0	53.7	45.9	31.5	28.3	76.8	74.0
9	55.7	26.5	57.6	75.4	33.0	28.7	77.6	73.2
10	56.2	56.8	59.6	51.5	34.3	30.5	78.4	76.1
11	56.9	40.6	59.3	58.2	34.4	26.7	78.6	72.3
12	55.9	62.8	57.2	33.6	32.9	26.2	77.6	70.7
13	54.8	42.9	57.2	70.4	32.4	35.2	77.1	76.7
14	56.5	73.8	57.8	42.2	33.4	32.4	78.0	81.1
15	58.0	53.1	52.9	48.2	31.6	27.8	77.1	73.3
Mean	56.1	51.9	57.2	54.5	33.0	30.3	77.6	75.1

Subject	F1-score (%)		G-Mean (%)		AUC-ROC (%)		AUC-PR (%)	
	Train	Test	Train	Test	Train	Test	Train	Test
1	39.8	41.6	55.1	57.5	57.5	58.5	33.5	35.2
2	42.0	40.7	57.3	56.4	60.3	60.0	35.8	33.2
3	41.6	40.9	56.8	46.0	59.2	53.1	34.4	30.3
4	41.4	36.4	56.6	51.0	59.4	50.9	34.6	28.1
5	40.8	36.0	55.2	52.5	57.3	55.9	32.8	30.8
6	42.6	32.4	57.9	48.7	61.2	50.5	36.6	27.1
7	42.1	42.2	57.4	58.0	60.5	61.2	36.1	37.0
8	40.5	37.8	55.2	51.1	57.3	52.3	32.8	28.2
9	41.4	27.6	56.6	44.7	59.2	52.1	34.4	29.5
10	42.6	39.7	57.9	54.1	61.0	55.4	36.5	31.9
11	42.9	32.2	58.1	48.6	61.5	48.7	36.5	27.5
12	41.4	37.0	56.6	45.9	59.2	48.4	34.3	27.5
13	40.7	38.7	56.0	54.9	58.6	59.0	33.7	36.0
14	42.0	45.0	57.2	55.8	60.3	63.0	35.3	37.0
15	40.9	36.5	55.4	50.6	57.6	50.6	33.1	27.7
Mean	41.5	37.6	56.6	51.7	59.3	54.6	34.7	31.1

Table 7. Sensitivity (Sen.), specificity (Spe.), Positive Predictive Value (PPV), Negative Predictive Value (NPV), F1-score, Geometric Mean (G-Mean), the area under the curve of the Receiver Operating Characteristics (AUC-ROC) and the area under the precision-recall curve (AUC-PR) for predicting *Fear* across the 15 subjects.

Subject	Sen (%)		Spe (%)		PPV (%)		NPV (%)	
	Train	Test	Train	Test	Train	Test	Train	Test
1	56.5	42.6	51.3	59.3	27.4	25.4	78.4	76.0
2	56.6	49.3	51.5	39.8	27.5	21.0	78.5	70.7
3	56.4	40.7	56.9	51.5	29.9	21.4	80.0	72.7
4	57.6	42.6	50.6	61.4	27.5	26.4	78.6	76.7
5	56.6	70.9	56.5	35.9	29.8	26.5	80.0	79.1
6	55.9	63.3	51.8	35.4	27.4	24.2	78.3	74.7
7	56.8	41.3	52.1	60.5	27.9	25.4	78.8	76.0
8	59.3	56.3	48.7	44.0	27.3	24.6	78.6	75.5
9	55.8	52.7	55.4	56.8	29.0	28.4	79.4	78.7
10	56.0	40.3	55.4	55.9	29.0	22.9	79.5	74.2
11	55.9	47.8	55.4	59.6	29.0	27.8	79.4	77.8
12	56.7	42.4	50.6	62.9	27.2	27.1	78.2	77.0
13	56.2	66.8	51.3	34.1	27.3	24.8	78.3	76.0
14	61.7	63.4	46.9	49.4	27.4	29.0	79.0	80.6
15	57.1	44.7	51.8	59.0	27.8	26.2	78.8	76.6
Mean	57.0	51.0	52.4	51.0	28.1	25.4	78.9	76.2

Subject	F1-score (%)		G-Mean (%)		AUC-ROC (%)		AUC-PR (%)	
	Train	Test	Train	Test	Train	Test	Train	Test
1	36.9	31.8	53.8	50.2	55.5	51.1	27.6	25.0
2	37.1	29.5	54.0	44.3	55.7	41.6	27.7	20.6
3	39.0	28.1	56.6	45.7	59.2	44.8	31.3	21.8
4	37.2	32.6	54.0	51.2	55.7	52.5	27.7	26.3
5	39.0	38.5	56.6	50.5	59.1	53.4	30.8	26.3
6	36.8	35.0	53.8	47.3	55.8	49.7	27.8	24.3
7	37.4	31.5	54.4	50.0	56.3	51.9	28.6	24.8
8	37.4	34.3	53.7	49.7	55.5	51.7	27.6	27.0
9	38.1	36.9	55.6	54.7	57.6	55.6	29.1	27.7
10	38.2	29.2	55.7	47.5	58.4	48.3	29.8	22.8
11	38.2	35.1	55.7	53.4	57.7	56.2	29.6	27.8
12	36.8	33.1	53.6	51.6	55.3	54.5	27.5	26.3
13	36.8	36.2	53.7	47.8	55.5	50.8	27.6	25.1
14	38.0	39.8	53.8	56.0	56.3	59.0	29.0	29.7
15	37.4	33.0	54.4	51.4	56.3	52.9	28.3	27.0
Mean	37.6	33.6	54.6	50.1	56.7	51.6	28.7	25.5

Table 8. Sensitivity (Sen.), specificity (Spe.), Positive Predictive Value (PPV), Negative Predictive Value (NPV), F1-score, Geometric Mean (G-Mean), the area under the curve of the Receiver Operating Characteristics (AUC-ROC) and the area under the precision-recall curve (AUC-PR) for predicting *Happy* across the 15 subjects.

Subject	Sen (%)		Spe (%)		PPV (%)		NPV (%)	
	Train	Test	Train	Test	Train	Test	Train	Test
1	55.3	49.5	57.6	51.4	25.9	21.4	82.8	79.2
2	56.4	65.6	57.8	60.5	26.4	30.8	83.2	86.8
3	59.0	22.7	61.3	83.5	29.0	26.8	84.8	80.1
4	55.9	43.3	58.6	72.2	26.6	29.4	83.2	82.6
5	56.9	65.0	58.6	37.5	26.9	21.8	83.5	80.0
6	58.4	79.0	58.6	50.8	27.4	30.1	84.0	90.0
7	58.9	53.7	59.9	51.6	28.2	22.9	84.5	80.6
8	55.1	47.6	57.6	58.2	25.8	23.4	82.7	80.6
9	56.3	62.0	59.2	42.8	27.0	22.5	83.5	80.8
10	54.2	53.3	58.7	63.6	26.0	28.2	82.7	83.6
11	58.0	11.0	59.1	78.9	27.5	12.2	84.0	76.8
12	56.2	39.9	57.9	83.9	26.3	39.8	83.2	83.9
13	57.0	69.0	58.5	37.1	26.9	22.7	83.6	81.7
14	59.1	59.5	60.2	56.5	28.5	26.8	84.6	83.9
15	57.3	46.5	58.2	60.3	26.9	23.9	83.6	80.8
Mean	56.9	51.2	58.8	59.2	27.0	25.5	83.6	82.1

Subject	F1-score (%)		G-Mean (%)		AUC-ROC (%)		AUC-PR (%)	
	Train	Test	Train	Test	Train	Test	Train	Test
1	35.3	29.9	56.5	50.5	58.8	51.5	27.2	24.6
2	35.9	41.9	57.1	63.0	60.4	68.2	29.0	37.3
3	38.8	24.6	60.1	43.5	63.8	62.9	32.5	26.1
4	36.0	35.0	57.2	55.9	60.9	60.4	29.7	27.1
5	36.5	32.6	57.7	49.4	61.3	52.7	29.6	24.6
6	37.3	43.5	58.5	63.3	62.1	70.6	31.0	37.0
7	38.1	32.1	59.4	52.6	63.2	55.7	32.2	29.6
8	35.2	31.4	56.3	52.7	58.9	54.0	27.5	22.6
9	36.5	33.0	57.7	51.5	61.2	54.2	29.7	23.7
10	35.1	36.9	56.4	58.2	59.0	60.6	27.4	29.9
11	37.3	11.6	58.5	29.4	62.1	41.0	30.2	17.3
12	35.9	39.8	57.0	57.8	60.2	70.0	29.1	39.3
13	36.5	34.2	57.8	50.6	61.1	57.5	29.4	29.7
14	38.4	37.0	59.7	58.0	63.3	62.7	32.6	28.1
15	36.6	31.6	57.7	53.0	61.4	53.4	29.8	24.4
Mean	36.6	33.0	57.8	52.6	61.2	58.4	29.8	28.1
